# Enteral Nutrition Combined with Improved-Sijunzi Decoction Shows Positive Effect in Precachexia Cancer Patients: A Retrospective Analysis

**DOI:** 10.1155/2021/7357521

**Published:** 2021-09-22

**Authors:** Yueying Li, Yajun Chen, Yaqi Zeng, Jie Dong, Chunlei Li, Yingjie Jia, Yonghua Zhao, Kun Wang

**Affiliations:** ^1^Department of Nutrition Therapy, Tianjin Medical University Cancer Institute and Hospital, National Clinical Research Center for Cancer, Tianjin, China; ^2^Key Laboratory of Cancer Prevention and Therapy, Tianjin, China; ^3^Tianjin's Clinical Research Center for Cancer, Huanhu West Road, Hexi District, Tianjin 300060, China; ^4^Department of Oncology, First Teaching Hospital of Tianjin University of Traditional Chinese Medicine, No. 88 Changling Road, Liqizhuang Street, Xiqing District, Tianjin 300380, China; ^5^State Key Laboratory of Quality Research in Chinese Medicine, Institute of Chinese Medical Sciences, University of Macau, Macau SAR, Taipa 999078, China

## Abstract

**Background:**

Cancer has been considered as the leading cause of death in the world. In patients with cancer, up to 80% display a cachectic period after diagnosis. Cachexia is known to have a negative impact on function, treatment tolerance, higher rates of hospitalizations, and mortality. Anorexia is often used as a warning sign of precachexia. Long-term anorexia may lead to malnutrition and, then, accelerate the occurrence of cachexia. A safe and effective treatment, which can both improve appetite and assist nutritional support for precachexia cancer patients shows its particular important role.

**Methods:**

A retrospective analysis comparing the different therapeutic effects on precachexia cancer patients with anorexia-malnutrition. We recorded 46 patients with the improved-Sijunzi decoction combined with enteral nutrition emulsion (ISJZ group) and 35 patients with single enteral nutrition emulsion (SEN group). The different therapeutic effects of the two groups were observed by recording indicators before and 2 weeks after treatment, including patient-generated subjective global assessment score, quality of life score, Karnofsky performance status scale, Eastern cooperative oncology group scale standard and traditional Chinese medicine syndrome, daily total dietary intake, red blood cells, hemoglobin, prealbumin, albumin, total protein cholinesterase, C-reactive protein, leukocytes, alanine aminotransferase, aspartate aminotransferase, alkaline phosphatase, urea nitrogen, and creatinine.

**Results:**

ISJZ group exhibited prominent improvement of traditional Chinese medicine syndrome (TCMS), nutritional condition, and quality of life compared with the SEN group (QOL: *p*=0.0001, PG-SGA: *p*=0.019, dietary intake: *p*=0.0001, TCMS: *p*=0.0001). The levels of HGB (*p*=0.006), PAlb (*p*=0.001), Alb (*p*=0.0001), TP (*p*=0.008), and ChE (*p*=0.0001) in the ISJZ group were higher than the SEN group after treatment. Moreover, the ratios of CRP/ALB (*p*=0.028) and CRP/PALB (*p*=0.005) in the two groups have obvious differences; they were lower for the ISJZ group than the SEN group.

**Conclusions:**

Enteral nutrition combined with ISJZ decoction is an effective treatment in precachexia cancer patients for the prevention of cachexia. This treatment therapy can alleviate the inflammatory response, improve malnutrition state, and promote the performance status. Tianjin Medical University Cancer Institute and Hospital approved this study (Trial No. 1913).

## 1. Introduction

Cancer has been considered as the leading cause of death in the world [1, 2]. Anorexia is one of the top three common symptoms in patients with cancer [3, 4], and it may induce malnutrition. The appearance of anorexia is often used as a warning sign of precachexia. It can be an independent prognostic factor for survival [5]. In patients with cancer, up to 80% display a cachectic period after diagnosis [6–8]. Cachexia is known to have a negative impact on function, treatment tolerance, higher rates of hospitalizations, and mortality [9, 10]. The development process of cachexia can be fast or slow, but it will all start from precachexia to refractory cachexia [7]; however, the reversal of refractory cachexia cannot be achieved at present. Hence, we should intervene in anorexia-malnutrition as soon as possible [11]. The treatment of anorexia-malnutrition includes invasive/parenteral nutrition support, enteral nutrition support, and appetite stimulants. However, we are still not sure which is the best time to offer artificial nutrition during cancer treatment [12]. What's more, some reports advised considering enteral and parenteral feeding only when the prognosis is longer than months to weeks [13]. The most extensively and successfully studied appetite stimulants are progestational agents and corticosteroids. Unfortunately, they all present side effects with varying degrees, such as the potential serious adverse events of megestrol acetate including thrombophlebitis [14] and adrenal insufficiency after abrupt drug withdrawal. Long-term steroids may induce metabolic changes, higher fracture rates, cataracts, and gastrointestinal discomfort [11]. Anamorelin is an oral ghrelin mimetic, and it has been proven to improve body weight. However, its drug toxicities included hyperglycemia and gastrointestinal disorders [15]. Therefore, we try to find a safe and effective treatment that can both improve appetite and assist nutritional support.

Sijunzi (SZJ) decoction comes from the Taiping Huimin and the Agent Bureau [16]. SJZ decoction has the effect of replenishing qi and invigorating spleen. It is used to treat the TCM syndrome of spleen qi deficiency. The main clinical manifestations of “spleen qi deficiency” include decreasing appetite, deficiency of food, debilitation, and abdominal distension (intermittently) [17, 18]. SJZ decoction could improve the immune function and QOL. What's more, SZJ decoction could maintain peripheral blood leukocyte count and peritoneal macrophage activity after chemotherapy; in addition, it could regulate the immune function of the body and enhance the anticancer effect of immune cells [19–22]. SJZ decoction could inhibit gastric cancer cell growth in vivo, and the mechanism was associated with the downregulation of the expression of p53 mRNA [23]. Chen et al. reported SJZ decoction produced anti-H22 tumor cells, upregulated the level of Bax, caspase-3, TNF-*α*, and IL-2, and downregulated Bcl-2 [24]. Dai et al. provided strong evidence for treating chronic fatigue syndrome by using SJZ decoction [25]. We improved the SJZ decoction to make it suitable for cancer patients with anorexia-malnutrition with spleen qi deficiency syndrome.

Some cancer patients with anorexia-malnutrition conform to the TCM syndrome of spleen qi deficiency syndrome. Long-term anorexia-malnutrition may lead to cachexia. In order to avoid the occurrence of irreversible cachexia, a safe and effective treatment urgently needs to be discovered, so we explored whether ISJZ decoction could have a positive effect on such patients. This passage reports the positive role of ISJZ in improving anorexia-malnutrition patients with spleen qi deficiency.

## 2. Materials and Methods

### 2.1. Study Population

We retrospectively reviewed the records of 81 patients between October 2018 and August 2020 in Tianjin Medical University Cancer Institute and Hospital. The study was conducted in accordance with the Declaration of Helsinki (as revised in 2013). The study was approved by ethics committee of Tianjin Medical University Cancer Institute and Hospital (No. E2019310). This study is a retrospective study and an informed consent form was not required. Inclusion criteria: (1) had pathologically diagnosed cancer; (2) age between 18–80 years old; (3) expected survival time was more than 3 months; (4) PG-SGA (patient-generated subjective global assessment) score ≥4 points; (5) 10 points < KPS (Karnofsky performance status) score ≤90 points; (6) 1 points ≤ ECOG (Eastern cooperative oncology group) score ≤4 points; (7) corresponded TCM syndromes of spleen qi deficiency; (8) corresponded the definition of “precachexia” [26]; and (9) patients accepted treatment by enteral nutritional support or ISJZ decoction combined with enteral nutritional support. Exclusion criteria: (1) need parenteral nutrition treatment; (2) ongoing anticancer treatment; (3) taking other Chinese herbal medicines or Chinese patent medicine in one week; (4) taking other drugs or blood products for treatment of malnutrition in one week; (5) had history of anti-infection therapies in one week; (6) had treatments that affected immune indicators; (7) taking appetite stimulants; (8) numeric pain rating scale score ≥7 points; (9) serious dysfunction of heart, lungs, brain, liver, or kidney; (10) confirmed state of refractory cachexia; and (11) disturbance of consciousness, dementia, or depression. We divided 81 patients into two groups according to the different treatment therapies, ISJZ group (*n* = 46) and SEN group (*n* = 35).

### 2.2. Assessments

We recorded the gender and age of each group of patients. The PG-SGA score, QOL score [27], KPS scale, ECOG scale standard, and TCM syndrome score [28, 29] (see the scoring method of traditional Chinese medicine syndrome in Table 1) were used to evaluate patients' subjective feelings and performance state. Daily total dietary intake, RBC (red blood cell), HGB (hemoglobin), PAlb (prealbumin), Alb (albumin), TP (total protein), and ChE (cholinesterase) were used to evaluate patients' objective nutritional status. We analyzed CRP (C-reactive protein) and WBC (leukocyte) to judge inflammation. ALT (alanine aminotransferase), AST (aspartate aminotransferase), ALP (alkaline phosphatase), UREA (urea nitrogen), and CREA (creatinine) were used to evaluate drug safety. All indicators were recorded before and 2 weeks after treatment to assess whether ISJZ decoction can have a positive effect on patients.

### 2.3. Treatment

SEN group: patients in this group were given enteral nutritional support to supply the gap of energy requirement (the gap of energy kcal = 30 kcal × weight (kg）- dietary intake (kcal)). Enteral nutrition emulsion TPF *T* (1.3 kcal/ml) was supplemented according to the calculation results for each patient.

ISJZ group: patients in this group were given ISJZ decoction, additionally, which consists of Codonopsis (dangshen) 30 g, rhizoma atractylodes (baizhu) 10 g, Poria cocos (fuling) 10 g, Radix glycyrrhizae (gancao) 6 g, Astragalus (huangqi) 30 g, Radix curcuma (jianghuang) 10 g, jiao Crataegus (shanzha) 10 g, jiao Medicated Leaven (shenqu) 10 g, and Ziziphus jujube (dazao) 10 g. Each component was processed into granules from Jiangyin Tianjiang Pharmaceutical Co, Ltd. (China). The patients were required to take the ISJZ granules twice a day (intake: mixed with hot water, 300 ml daily, taken morning and evening, 150 ml each time). The basic nutrition treatment was the same as that of the SEN group.

### 2.4. Statistical Analysis

SPSS software (Version 23.0, SPSS Inc, Chicago, IL, USA) was used for statistical analysis. The Shapiro–Wilk test was used to detect whether the data conform to the normal distribution. Normally distributed data were expressed as mean ± standard deviation （*X*± SD）, using the *T* test to compare two groups of data. Abnormal distributed data were expressed as median (p25, p75), using Mann–Whitney *U* test to compare the two groups of data. The categorical data were expressed as ratios (%), using Chi-square tests to compare between groups. *p* < 0.05 (two-sided) was recorded as a statistically significant difference between the two groups.

## 3. Results

### 3.1. Characteristics of Patients

A total of 81 patients were included in our study, and all of them belong to clinical stage IV.  Table 2 detailed the cancer primary site of 2 groups of patients. Patients in 2 groups had similar age (58.7 ± 11.3 vs. 63.1 ± 9.8) and sex (male: 52.17% vs. 60%) composition, and there was no statistical difference in the type of previous treatment (1.98 ± 0.99 vs. 2.15 ± 1.18; previous treatment included operation, radiotherapy, chemotherapy, hormonal treatment, targeted therapy, and immunotherapy). However, the analysis showed that the number of distant metastasis between the two groups was statistically significant (*p*=0.026). In the comparison of the baseline nutritional status, there were no significant differences in PG-SGA, QOL, KPS, ECOG, and TCMS scores between the two groups (Table 2).

### 3.2. Performance Status, Nutritional Condition, and Quality of Life Assessment

Before treatment, patients in the two groups were in high nutritional risk and urgently needed proper nutritional support. After treatment, the nutritional condition of the patients had improved to varying degrees in the two groups. The change in the PG-SGA score and TCMS score in the ISJZ group was decreased more than that in SEN group. The change in the dietary intake and QOL score in the ISJZ group was increased more than that in the SEN group, they exhibited a significant difference in the 2 groups (*p*=0.0001, Table 3). The difference in the KPS and ECOG scores had no significant difference between the 2 groups. The results demonstrated that enteral nutrition could improve patients' quality of life and alleviate clinical symptoms. This when combined with ISJZ decoction could amplify the effect.

### 3.3. Hematological Examination

Next, we compared the changes in hematologic examination before and after the treatment of patients in the 2 groups (Table 4). The analysis showed that the RBC, HGB, PAlb, Alb, TP, and ChE levels were increased at the end of treatment in the ISJZ group. However, the RBC, HGB, PAlb, Alb, and TP levels were decreased at the end of treatment in the SEN group. There was no difference in HGB, PAlb, Alb, TP, and ChE levels between the two groups before treatment, but there was a significant difference at the end of treatment (*p*=0.006,  *p*=0.001,  *p*=0.0001,  *p*=0.008,  *p*=0.0001) and the difference was statistically significant (*p*=0.0001, *p*=0.0001, *p*=0.0001, *p*=0.0001, *p*=0.043).

Furthermore, the analysis showed that the CRP level was decreased at the end of treatment in two groups. The levels of CRP/Alb and CRP/PAlb were decreased in the ISJZ group; however, they were increased in the SEN group at the end of the treatment. There was no difference in CRP/Alb and CRP/PAlb levels between the two groups before treatment, but there was a significant difference at the end of treatment (*p*=0.028,  *p*=0.005) and the difference was statistically significant (*p*=0.027 and *p*=0.032). There was no difference in the level of WBC between the two groups before and after treatment.

### 3.4. Evaluation of Drug Safety

We evaluated the drug safety by monitoring the liver and kidney functions. ALT, AST, ALP, CREA, and UREA were selected to observe whether the drug has liver or kidney toxicity. The degrees of ALT, AST, ALP, and CREA showed no significant differences before and after treatment in two groups. This result suggested that ISJZ decoction had no obvious liver and kidney toxicity.

## 4. Discussion

Cancer anorexia is diagnosed on the basis of loss of appetite, and/or early satiety, taste alterations, nausea, and vomiting [30], which caused energy intake deficiency [31]. Continuous energy intake deficiency may lead to patient malnutrition, cause serious effects on patient's quality of life, and interfere with cancer treatment therapy. The formation of cachexia can be attributed to two factors: one is tumor-induced metabolic changes and another is anorexia-induced malnutrition [30, 32]. We observed peripheral blood samples as a prognostic indicator for judging patients with malignant tumors [33].

Multiple inflammatory responses play a pivotal part in tumor-induced metabolic changes. The ratio CRP/Alb is a prognostic indicator for several cancers [34]. Because of its shorter half-life, PAlb is used as a good indicator of visceral protein [35]. Some researchers suggested that CRP/PAlb was more sensitive to CRP/Alb for cancer prognosis. High CRP/Alb or CRP/PAlb ratio was significantly associated with bad prognosis [36]. Our results showed that patients who only accepted enteral nutrition had a decreasing trend in the CRP level, but not in the ratio of CRP/Alb and CRP/PAlb. However, the levels of CRP, CRP/Alb, and CRP/PAlb in patients who underwent treatment in combination with ISJZ decrease at the same time. So far, we have boldly assumed that ISZJ decoction could alleviate the inflammatory response triggered by cancer, so that it partly suppressed the consequence of metabolic abnormalities.

Anorexia-induced malnutrition has a negative effect on prognosis. ChE as a nutrition-related serum marker is useful for predicting the prognosis of patients with several types of cancer [37, 38]. Patients with low levels of ChE and Alb have the lowest 5-year overall survival rates than those patients with high levels of ChE and Alb or high ChE/low Alb or low ChE/high Alb [37]. Our results indicated that the short-term enteral nutritional treatment failed to increase the level of ChE or Alb. However, treatment combined with ISJZ decoction could increase the ChE level (6340.07 ± 1594.36) and the Alb (36.22 ± 5.04) level (Table 4). Our results also showed that the levels of RBC, HGB, PAlb, and TP were increased within the normal range in the ISJZ group. Additionally, the value of dietary intake in the SEN group was increased lower than that in the ISJZ group, and the score of TCMS in the SEN group was decreased lower than that in the ISJZ group. On the basis of these data, we speculated that the reason why only accepted short-term enteral nutrition could not improve the nutritional status in time might be related to its inability to improve appetite and absorption. The addition of ISJZ on the basis of nutritional therapy could effectively increase appetite and improve nutritional status, thereby might have an opportunity to increase the five-year survival rate.

As a predicted survival in patients with cancer, the performance status (PS) had the ability to measure the activities of daily living. It is well known that patients with poor performance status always avoid more aggressive treatment [39–41]. The KPS score and ECOG score are the most commonly used for the assessment of PS. Generally speaking, the KPS score is required no less than 70, and the ECOG score is required no more than 2 before relevant anticancer treatments are considered. We recorded two groups of patients, KPS and ECOG scores. Our results showed that KPS score was less than 70 and ECOG score was more than 2 in both groups before treatment. It is demonstrated that the conditions of patients were suggested not to tolerate more aggressive anticancer treatment. After treatment, KPS scores of the two groups were all increased, and the ECOG scores of the two groups were all decreased. Regrettably, we could not find that ISJZ had a significant advantage than SEN in a short treatment period. However, it is worth noting that the score of KPS and ECOG in the ISJZ group had significant differences before and after treatment, especially the score of ECOG decreased to lower than 2(1.87 ± 0.45, Table 3). The TCMS score of the ISJZ group was significantly lower than that of the SEN group (*p*=0.0001, Table 3), the QOL score of the ISJZ group was significantly higher than that in the SEN group (*p*=0.0001, Table 3), and the differences were statistically significant after treatment. Hence, we thought that ISJZ decoction can promote the physical recovery of patients, reduce the clinical symptoms of patients, and reduce the suffering of patients.

Our study has certain limitations. First, the study was not a double-blind random trial. Second, the term of observation was short, so we could not give the evaluation of long-term effects. Third, our current evidence could only show that ISJZ decoction was suitable for patients with spleen-qi deficiency. Nevertheless, our results gave strong evidence for the possibility that enteral nutrition combined with ISJZ decoction was efficacious and safe for improving precachexia in cancer patients with spleen qi deficiency.

## 5. Conclusions

Our findings suggested that the treatment of enteral nutrition combined with ISJZ decoction could (i) alleviate the inflammatory response triggered by cancer, (ii) improve the malnutrition state triggered by anorexia, and (iii) promote patient's performance status.

## Figures and Tables

**Table 1:** The scoring method of traditional Chinese medicine syndrome.

Symptom	1 score	2 score	3 score
Decreased food intake	Less than 1/3	Between 1/3 and 1/2	More than ½
Lack of strength	Unable to adhere manual labor	Barely stick to daily activities	Unable to adhere to daily activities
Abdominal distention	Mild fullness, abdominal distension after eating, relieved within half an hour.	Abdominal distension, obvious after eating, relieved within half an hour to 1 hour.	Abdominal distension is obvious, especially after eating, relieving takes more than 1 hour.

Constipation or loose stools	1 score		

TCMS score was the sum of each symptom score.

**Table 2:** Characteristic of patients.

Characteristic	ISJZ group (*n* = 46), *n* (%)	SEN group (*n* = 35), *n* (%)	*p*-value
Male	24 (52.17)	21 (60)	0.221
Female	22 (47.83)	14 (40)	
Age (years)	58.7 ± 11.3	63.1 ± 9.8	0.388
PG-SGA score	15.48 ± 5.16	15.23 ± 4.37	0.819
QOL score	25.26 ± 4.07	23.31 ± 6.1	0.089
KPS score	59.13 ± 8.90	60.29 ± 7.47	0.537
ECOG score	2.02 ± 0.61	2.11 ± 0.63	0.509
TCMS score	5.74 ± 1.45	5.86 ± 1.35	0.710

Primary site			
Bile duct	1	0	—
Bladder	1	1	—
Breast	4	4	—
Cervix	4	3	—
Esophagus	5	6	—
Kidney	1	1	—
Liver	1	1	—
Lung	18	7	—
Ovaries	2	0	—
Pancreas	3	3	—
Prostate	0	3	—
Rectum	1	2	—
Stomach	0	2	—
Ureter	0	1	—
Others	5	1	—
Number of distant metastasis	2.02 ± 1.14	1.56 ± 0.66	0.026^*∗*^
Type of previous treatment	1.98 ± 0.99	2.15 ± 1.18	0.293

^*∗*^, statistical significance at *p* < 0.05. PG-SGA, patient-generated subjective global assessment; QOL, quality of life; KPS, Karnofsky performance status; ECOG, Eastern cooperative oncology group; TCMS: traditional Chinese medicine syndrome.

**Table 3:** Comparison of patients' quality of life and nutritional status.

Survey score	Time	ISJZ group	SEN group	*p*-value
PG-SGA score	Baseline	15.48 ± 5.16	15.23 ± 4.37	0.819
	Ending	7.33 ± 2.16	13.09 ± 3.88	0.019^*∗*^
	Difference	−8.50 (−11.25, −4.75)	−2.50 (−2.75, 0.00)	0.0001^*∗∗*^

Dietary intake (kcal)	Baseline	980 ± 113	1014 ± 123	0.198
	Ending	1284 ± 122	1017 ± 92	0.0001^*∗∗*^
	Difference	293.00 (208.50, 453.50)	31.00 (−90.00, 125.00)	0.0001^*∗∗*^

QOL score	Baseline	25.26 ± 4.07	23.31 ± 6.1	0.089
	Ending	37.04 ± 3.15	28.40 ± 4.68	0.0001^*∗∗*^
	Difference	11.00(9.75, 14.00)	6.00 (3.00, 7.00)	0.0001^*∗∗*^

KPS score	Baseline	59.13 ± 8.90	60.29 ± 7.47	0.537
	Ending	62.17 ± 5.93	61.43 ± 6.48	0.592
	*p*-value^*∗∗∗*^	0.0001^*∗∗*^	0.074	
	Difference	0.00 (0.00, 10.00)	0.00 (0.00, 0.00)	0.093

ECOG score	Baseline	2.02 ± 0.61	2.11 ± 0.63	0.509
	Ending	1.87 ± 0.45	2.00 ± 0.59	0.265
	*p*-value^*∗∗*^	0.003^*∗*^	0.659	
	Difference	0.00 (0.00, 0.00)	0.00 (0.00, 0.00)	0.686

TCMS score	Baseline	5.74 ± 1.45	5.86 ± 1.35	0.710
	Ending	4.22 ± 1.07	5.26 ± 1.04	0.0001^*∗∗*^
	Difference	−1.00 (−2.00, −1.00)	−1.00 (−1.00, −1.00)	0.01^*∗*^

^*∗*^Statistical significance at *p* < 0.05; ^*∗∗*^statistical significance at *p* < 0.001. Baseline and ending: mean ± SD; difference: median (p25, p50). ^*∗∗∗*^*p*-value between baseline and ending in the same group. PG-SGA, patient enerated subjective global assessment; QOL, quality of life; KPS, Karnofsky performance status; ECOG, Eastern Cooperative Oncology Group; TCMS: traditional Chinese medicine syndrome.

**Table 4:** Comparison of patients' blood indicators.

Blood indicator	Time	ISJZ group	SEN group	*p*-value
RBC (10^12/L)	Baseline	3.54 ± 0.60	3.98 ± 0.69	0.004^*∗*^
	Ending	3.81 ± 0.58	3.55 ± 0.76	0.092
	Difference	0.21 (0.10, 0.45)	−0.35 (−0.62, −0.18)	0.0001^*∗∗*^

HGB (g/L)	Baseline	106.48 ± 16.47	114.60 ± 20.36	0.051
	Ending	114.17 ± 17.43	101.91 ± 20.50	0.006^*∗*^
	Difference	7.00 (2.00, 12.00)	−10.00 (−20.00, −5.00)	0.0001^*∗∗*^

PAlb (g/L)	Baseline	0.14 ± 0.05	0.14 ± 0.07	0.638
	Ending	0.19 ± 0.07	0.13 ± 0.07	0.001^*∗∗*^
	Difference	0.05 (0.02, 0.083)	−0.02 (−0.07, 0.06)	0.0001^*∗∗*^

Alb (g/L)	Baseline	32.63 ± 6.89	35.44 ± 9.24	0.120
	Ending	36.22 ± 5.04	30.24 ± 6.64	0.0001^*∗∗*^
	Difference	2.85 (0.70, 4.90)	−2.6 ( 7.05, 0.13)	0.0001^*∗∗*^

TP (g/L)	Baseline	61.15 ± 6.96	66.76 ± 7.21	0.053
	Ending	67.13 ± 7.15	62.06 ± 8.97	0.008^*∗*^
	Difference	4.95 (2.65, 7.68)	−4.90 (−9.50, 3.50)	0.0001^*∗∗*^

ChE (U/L)	Baseline	5671.21 ± 1708.04	4930.71 ± 2053.72	0.081
	Ending	6340.07 ± 1594.36	4776.43 ± 1893.46	0.0001^*∗∗*^
	Difference	576.50 (93.50, 890.50)	61.00 (−2055.00, 827.00)	0.043^*∗*^

CRP (mg/L)	Baseline	42.75	37.80	0.535
	Ending	19.75	38.10	0.081
	Difference	−10.27 (−45.99, 2.38)	−3.00 (−41.60, 14.00)	0.308

CRP/Alb	Baseline	1.32 (0.28, 2.69)	0.96 (0.52, 2.94)	0.696
	Ending	0.57 (0.14, 1.67)	1.52 (0.41, 3.16)	0.028^*∗*^
	Difference	−0.38 (−1.76, 0.014)	0.045 (−0.92, 1.32)	0.027^*∗*^

CRP/PAlb	Baseline	0.33 (0.07, 0.93)	0.46 (0.10, 1.04)	0.443
	Ending	0.11 (0.03, 0.34)	0.38 (0.11, 0.94)	0.005^*∗*^
	Difference	−0.14 (−0.58, −0.00)	0.02 (−0.42, 0.37)	0.032^*∗*^

WBC (10^9/L)	Baseline	7.40 ± 3.61	9.06 ± 4.37	0.065
	Ending	7.55 ± 3.45	8.21 ± 3.80	0.413
	Difference	0.56 (−0.33, 1.92)	−0.80 (−2.24, 0.60)	0.01^*∗*^

ALT (U/L)	Baseline	19.98 ± 15.34	18.71 ± 14.60	0.709
	Ending	27.32 ± 17.42	20.29 ± 16.06	0.066
	Difference	3.00 (−4.25, 18.75)	1.00 (−13.00, 11.00)	0.256

AST (U/L)	Baseline	24.54 ± 13.58	30.60 ± 25.02	0.167
	Ending	29.54 ± 15.81	32.86 ± 32.86	0.551
	Difference	2.50 (−3.50, 12.25)	−2.00 (−14.00, 17.00)	0.190

ALP (U/L)	Baseline	125.74 ± 76.38	154.09 ± 114.33	0.185
	Ending	140.48 ± 79.65	189.91 ± 206.49	0.141
	Difference	8.00 (−3.75, 24.50)	3.00 (−64.00, 73.00)	0.706

CREA (*μ*mol/L)	Baseline	55.93 ± 11.97	56.51 ± 16.77	0.856
	Ending	52.56 ± 11.80	51.34 ± 16.63	0.704
	Difference	−1.00 (−7.5, 1.50)	−5.00 (−24.00, 8.00)	0.464

UREA (mmol/L)	Baseline	4.52 ± 1.48	6.36 ± 4.68	0.030^*∗*^
	Ending	4.97 ± 1.93	5.73 ± 2.39	0.116
	Difference	0.20 (−0.45, 1.00)	−0.10 (−3.10, 1.70)	0.167

^*∗*^Statistical significance at *p* < 0.05; ^*∗∗*^statistical significance at *p* < 0.001. Baseline and ending: mean ± SD; difference: median (p25, p50). RBC, red blood cell; HGB, hemoglobin; PAlb, prealbumin; Alb, albumin; TP, total protein; ChE, cholinesterase; CRP, C-reactive protein; WBC, leukocyte; ALT, alanine aminotransferase; AST, aspartate aminotransferase; ALP, alkaline phosphatase; CREA, creatinine; UREA, urea nitrogen.

## Data Availability

The data in this study are available on request from the corresponding author. The data are not publicly available due to patient privacy.

## Abbreviations

Alb: Albumin
ALP: Alkaline phosphatase
ALT: Alanine aminotransferase
AST: Aspartate aminotransferase
ChE: Cholinesterase
CRP: c-Reactive protein
CREA: Creatinine
ECOG: Eastern Cooperative Oncology Group
HGB: Hemoglobin
KPS: Karnofsky performance status
PAlb: Prealbumin
PG-SGA: Patient-generated subjective global assessment
PS: Performance status
QOL: Quality of life
RBC: Red blood cells
TCMS: Traditional Chinese medicine syndrome
TP: Total protein
UREA: Urea nitrogen
WBC: Leukocyte.
